# Acknowledgement to Reviewers of *Vision* in 2018

**DOI:** 10.3390/vision3010006

**Published:** 2019-01-28

**Authors:** Vision Editorial Office

**Affiliations:** MDPI, St. Alban-Anlage 66, 4052 Basel, Switzerland

Rigorous peer-review is the corner-stone of high-quality academic publishing. The editorial team greatly appreciates the reviewers who contributed their knowledge and expertise to the journal’s editorial process over the past 12 months. In 2018, a total of 42 papers were published in the journal, with a median time to first decision of 21.5 days and a median time to publication of 53.5 days. The editors would like to express their sincere gratitude to the following reviewers for their cooperation and dedication in 2018:

Amoaku, Winfried M.Kremers, JanAshida, HiroshiKuriki, IchiroAznar-Casanova, Jose AntonioLabhishetty, VivekBaker, Daniel H.Lages, MartinBarrett, DougLaidlaw, KaitlinBedell, HaroldLappin, Joseph S.Bengson, Jesse J.Laurent, EricBernhardt-Walther, DirkLevy, StevenBinda, PaolaLiu, ChunmingBosten, JennyMallen, EdwardBrooks, Kevin R.Marini, FrancescoBrooks, JosephMather, GeorgeBruno, AurelioMatthews, NestorTheobald, Jamie C.McClelland, JulieCameron, MorvenMeduri, AlessandroCapozzi, FrancescaMeier, KimberlyCarroll, Lara S.Morikawa, KazunoriCass, JohnMüller, HermannCastet, EricMunaron, LucaChang, DoritaNebbioso, MarcellaChen, JuanOdom, VernonChen, Chien-ChungOuhnana, MarouaneChristie, JohnPanorgias, ThanasisCollins, ThérèsePenacchio, OlivierCoppola, GianlucaPepperell, RobertCufflin, MattPilling, MichaelCumming, BrucePinto, YaïrDas, Vallabh E.Poletti, MartinaDavidenko, NicolasPratte, MichaelD'Avossa, GiovanniPtak, RadekDe Castro Arribas, AlbertoQuaranta, LucianoDe Graaf, Tom AlexanderRaidvee, AireDe Haan, E.H.F. (Edward)Rambold, Holger A.Deska, JasonRead, JennyDeSouza, Joseph FrancisReeves, AdamDresp-Langley, BirgittaRichard, BrunoDurgin, Frank H.Riva, IvanoErkelens, CasperRoberts, TawnaEssock, EdwardRogers, Jeremy D.Fahle, ManfredRossi, Ethan A.Fautsch, Michael P.Saint-Amour, DaveFelisberti, Fatima M.Santana-Mancilla, Pedro C.Fortenbaugh, FrancescaSantos, Jesús DanielFrancis, GregorySawada, TadamasaFranco, SandraSchallmo, Michael-PaulFreeman, AlanSchmid, MichaelFrench, RobertSedgwick, H. A.Galati, AlexiaSegev, RonenGeorgeson, MarkSengpiel, FrankGherri, ElenaSerres, JulienGiacomelli, GiovanniShepherd, AlexGiovingo, Michael CarloShu, XinhuaGonzález, EstherSlessor, GillianGordon, GaelSmith, DanielGrabowecky, MarciaStepanova, ElenaHaak, Koen V.Stewart, Michael W.Hahn, BrittaSubhi, YousifHarrison, Neil R.Taylor, ChristopherHarvey, BenThompson, Richard B.Haun, AndrewTodd, James T.Hess, Robert F.Topolinski, SaschaHills, Peter J.Tran, Thi Ha ChâuHutchinson, ClaireTripathy, SrimantIrwin, DavidVan Buren, BenJabar, SyaheedVessey, KirstanJack, BradleyWallis, StuartJones, AlexanderWard, JamieKelly, Jonathan W.Whitford, VeronicaKhamis, MohamedWijesinghe, Ruchire ErangaKlink, ChrisWuerger, SophieKosovicheva, Anna A.Ziemssen, FockeAmoaku, Winfried M.Kremers, Jan

